# Investigating the Comparative Effectiveness of Place-Based and Scatter-Site Permanent Supportive Housing for People Experiencing Homelessness During the COVID-19 Pandemic: Protocols for a Mixed Methods, Prospective Longitudinal Study

**DOI:** 10.2196/46782

**Published:** 2023-04-28

**Authors:** Benjamin F Henwood, Randall Kuhn, Howard Padwa, Roya Ijadi-Maghsoodi, Gisele Corletto, Alex Lawton, Jessie Chien, Ricky Bluthenthal, Michael R Cousineau, Melissa Chinchilla, Bikki Tran Smith, Katherine D Vickery, Taylor Harris, Maria Patanwala, Whitney Akabike, Lillian Gelberg

**Affiliations:** 1 Suzanne Dworak-Peck School of Social Work University of Southern California Los Angeles, CA United States; 2 Department of Community Health Sciences Fielding School of Public Health University of California Los Angeles, CA United States; 3 Semel Institute for Neuroscience and Human Behavior University of California Los Angeles, CA United States; 4 Center for the Study of Healthcare Innovation, Implementation and Policy Veterans Affairs Greater Los Angeles Healthcare System Los Angeles, CA United States; 5 Department of Population and Public Health Sciences Keck School of Medicine University of Southern California Los Angeles, CA United States; 6 Department of Biomedical and Health Sciences College of Nursing and Health Sciences University of Vermont Burlington, VT United States; 7 University of Minnesota Medical School Minneapolis, MN United States; 8 Health, Homelessness, and Criminal Justice Lab Hennepin Healthcare Research Institute Minneapolis, MN United States; 9 Department of Family Medicine David Geffen School of Medicine University of California Los Angeles, CA United States; 10 Department of Health Policy and Management Fielding School of Public Health University of California Los Angeles, CA United States

**Keywords:** homelessness, housing first, permanent supportive housing, COVID-19, homeless, housing, health outcome, patient-centered, person-centered, photo-elicitation

## Abstract

**Background:**

Permanent supportive housing (PSH) is an evidence-based practice to address homelessness that is implemented using 2 distinct approaches. The first approach is place-based PSH (PB-PSH), or single-site housing placement, in which an entire building with on-site services is designated for people experiencing homelessness. The second approach is scatter-site PSH (SS-PSH), which uses apartments rented from a private landlord while providing mobile case management services.

**Objective:**

This paper describes the protocols for a mixed methods comparative effectiveness study of 2 distinct approaches to implementing PSH and the patient-centered quality of life, health care use, and health behaviors that reduce COVID-19 risk.

**Methods:**

People experiencing homelessness who are placed in either PB-PSH or SS-PSH completed 6 monthly surveys after move-in using smartphones provided by the study team. A subsample of participants completed 3 qualitative interviews at baseline, 3 months, and 6 months that included photo elicitation interviewing. Two stakeholder advisory groups, including one featuring people with lived experience of homelessness, helped guide study decisions and interpretations of findings.

**Results:**

Study recruitment was supposed to occur over 6 months starting in January 2021 but was extended due to delays in recruitment. These delays included COVID-19 delays (eg, recruitment sites shut down due to outbreaks and study team members testing positive) and delays that may have been indirectly related to the COVID-19 pandemic, including high staff turnover or recruitment sites having competing priorities. In end-July 2022, in total, 641 people experiencing homelessness had been referred from 26 partnering recruitment sites, and 563 people experiencing homelessness had enrolled in the study and completed a baseline demographic survey. Of the 563 participants in the study, 452 had recently moved into the housing when they enrolled, with 272 placed in PB-PSH and 180 placed in SS-PSH. Another 111 participants were approved but are still waiting for housing placement. To date, 49 participants have been lost to follow-up, and 12% of phones (70 of the initial 563 distributed) were reported lost by participants.

**Conclusions:**

Recruitment during the pandemic, while successful, was challenging given that in-person contact was not permitted at times either by program sites or the research institutions during COVID-19 surges and high community transmission, which particularly affected homelessness programs in Los Angeles County. To overcome recruitment challenges, flexible strategies were used, which included extending the recruitment period and the distribution of cell phones with paid data plans. Given current recruitment numbers and retention rates that are over 90%, the study will be able to address a gap in the literature by considering the comparative effectiveness of PB-PSH versus SS-PSH on patient-centered quality of life, health care use, and health behaviors that reduce COVID-19 risk, which can influence future public health approaches to homelessness and infectious diseases.

**Trial Registration:**

ClinicalTrials.gov NCT04769349; https://clinicaltrials.gov/ct2/show/NCT04769349

**International Registered Report Identifier (IRRID):**

DERR1-10.2196/46782

## Introduction

The COVID-19 pandemic has made housing and health care for people experiencing homelessness an important priority for the health care and public health systems in the United States. Due to this population’s preexisting vulnerabilities [1-3] and difficulties in maintaining social distance and hygiene, researchers predicted at the outset of the COVID-19 pandemic that, without intervention, people experiencing homelessness would experience a 2-4 times higher risk of COVID-19 complications and fatality than the general population [4]. As a result of these concerns, more than US $2 billion from the US federal government’s Coronavirus Aid, Relief, and Economic Security Act was aimed at protecting people experiencing homelessness by reducing shelter densities and placing people in more secure housing options.

Permanent supportive housing (PSH)—programs that provide immediate access to independent living situations coupled with support services—represent one of the most studied and effective approaches for serving highly vulnerable people experiencing homelessness [5]. Numerous studies have demonstrated PSH’s effectiveness in improving housing retention, quality of life, and HIV outcomes. Nevertheless, a 2018 report by the National Academy of Medicine of the National Academies of Sciences noted the need for more research on PSH to establish what would constitute “housing-sensitive [health] conditions”—conditions whose transmissibility, course, and medical management are particularly influenced by homelessness and housing placement [5]. Two years after this report, COVID-19 and rapid policy decisions to address homelessness and expand housing during the pandemic offered a rare opportunity to address this gap in knowledge and advance research on housing as a social determinant of health.

This paper describes the protocols for the Person-Centered Housing Options, Outcomes, Services, and Environment (PCHOOSE) study, which examined the comparative effectiveness of 2 distinct approaches to implementing PSH. The first approach is place-based PSH (PB-PSH), or single-site housing placement, in which an entire building with on-site services is designated for people experiencing homelessness. The second approach is scatter-site PSH (SS-PSH), which uses apartments rented from a private landlord while providing mobile case management services. The limited research related to the comparative effectiveness of PB-PSH versus SS-PSH has been mixed. A meta-analysis of 8 studies with more than 3000 participants found 84% of people experiencing homelessness with mental disorders preferred SS-PSH [6], despite greater feelings of social isolation than those in PB-PSH. This is supported by qualitative research that has found people experiencing homelessness dislike the rules and limited privacy of PB-PSH [6-8]. Other studies have suggested PB-PSH may provide more supportive services [9] and may be more effective than SS-PSH in improving disability severity, community integration, and recovery [10]. Individuals with substance use disorders, those living with HIV, veterans, and women may also benefit more from PB-PSH programs than other populations because of the strong sense of community and mutual support they offer [11]. The PCHOOSE study intended to add to this literature by considering the comparative effectiveness of PB-PSH versus SS-PSH on patient-centered quality of life, health care use, and health behaviors that reduce COVID-19 risk.

## Methods

### Setting and Conceptual Model

**Figure 1:**
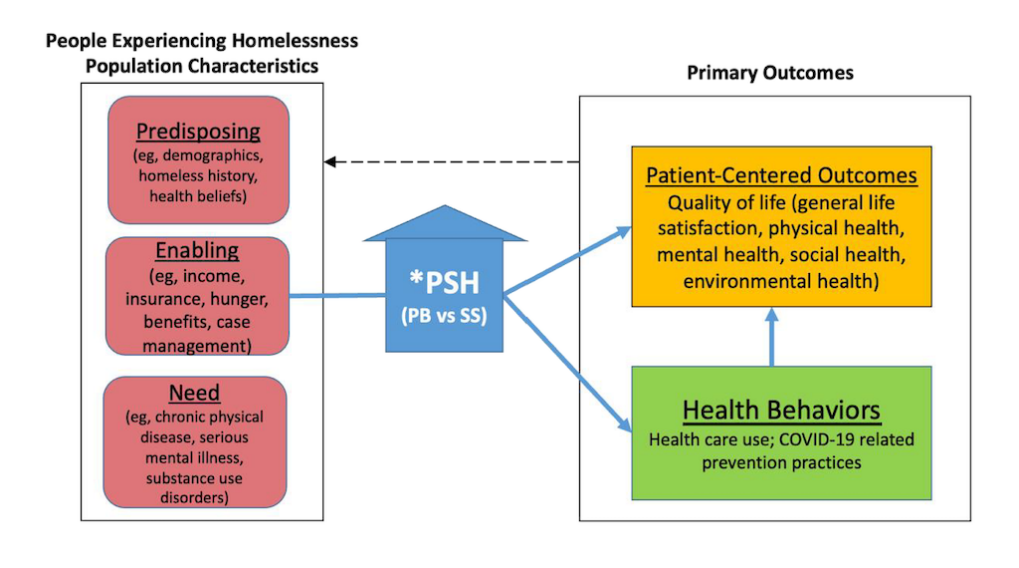
Gelberg-Anderson behavioral model for vulnerable populations (modified for PSH and COVID-19). PB: place-based; PSH: permanent supportive housing; SS: scatter-site.

The PCHOOSE study is situated in Los Angeles County, California, which has the nation’s largest unsheltered homeless population [12]. Los Angeles County also represents the country’s largest natural experiment in housing interventions to protect people experiencing homelessness [13] due to a major taxpayer-funded US $1.2 billion homelessness initiative passed in 2016 that resulted in the opening of thousands of new PSH units in Los Angeles County during the pandemic [14]. The study followed a diverse sample of people experiencing homelessness placed in either PB-PSH or SS-PSH for 6 months; barriers and facilitators that may affect PSH implementation during the pandemic and its aftermath were also investigated. The study was guided by the Gelberg-Anderson behavioral model for vulnerable populations [15], which involves examining predisposing, enabling, and need factors that contribute to health behaviors and patient-centered outcomes (see Figure 1). PSH is positioned as a key enabling factor for people experiencing homelessness with the hypothesis that PSH type (ie, PB or SS) will affect THE quality of life (general life satisfaction; physical, mental, social, and environmental health, including housing and neighborhood characteristics) and health behaviors, including health care use (eg, receipt of health care, mental health care, and social supports) and COVID-19–related prevention practices. We also expected that changes to our primary outcomes would affect the predisposing, enabling, and need characteristics of the people experiencing homelessness population over time.

In describing the protocols of the PCHOOSE study, this paper also highlights the challenges of conducting research with this vulnerable population in the United States during the highly dynamic COVID-19 pandemic. This includes steps taken to protect the research team, people experiencing homelessness participants, and PSH staff members, as well as the need to change survey instruments to reflect an evolving understanding of the COVID-19 virus, public health mitigation policies, and personal protective practices.

### Design Overview

This study leveraged Los Angeles County’s ongoing efforts to provide PSH to people experiencing homelessness during the COVID-19 pandemic using a mixed methods, prospective longitudinal design. We recruited people moving into PSH and provided them with smartphones with paid data plans to stay connected with the study for a 6-month period. All study participants were recruited through organizations that provide housing, supportive services, or both. Although PB-PSH is clearly defined in the homelessness services system, SS-PSH could have been officially categorized as a PSH program or a rapid rehousing program. Although rapid rehousing programs are typically short-term housing programs with time-limited and tailored assistance programs, during the pandemic, Los Angeles County expanded the use of rapid rehousing programs coupled with supportive services to quickly provide access to housing. Thus, for this study, we considered anyone receiving rapid rehousing assistance to be SS-PSH if they also received support services, and examined whether those receiving rapid rehousing differ in terms of demographics, health status, or homelessness histories.

For the study’s quantitative arm, participants completed a baseline survey followed by 5 monthly follow-up surveys once in housing. For the qualitative arm, we recruited a purposive subsample of people experiencing homelessness from both PB-PSH and SS-PSH who participated in 3 semistructured interviews; the first was an in-depth interview when they initially moved into PSH, followed by 2 follow-up interviews conducted 3 and 6 months later. Focus groups with housing providers from the affiliated housing agencies were also conducted. Study protocols for each study component are described separately in more detail.

To ensure that the study addressed questions important to people experiencing homelessness, providers, and policy makers and that the data were correctly interpreted, we also established 2 stakeholder advisory boards (SABs). The first advisory board is a lived experience group (LEG), which consists of 11 individuals who have personally experienced homelessness. The LEG meets quarterly and uses its lived experience, knowledge of what is important to people experiencing homelessness, and perspectives on how people experiencing homelessness may respond to study questions to inform recruitment strategies, interpretation of study findings, and dissemination efforts. The study’s second advisory board is a SAB, which consists of 15 providers, administrators, policy makers, and researchers with expertise in homelessness and housing programs in Los Angeles County. Due to concerns about meeting in person during the pandemic, all LEG and SAB meetings have been conducted remotely via Zoom.

### Ethics Approval

This human subjects’ research has been performed in accordance with the Declaration of Helsinki and approved by the University of Southern California’s Institutional Review Board (UP-20-01081) with a reliance agreement in place with the University of California, Los Angeles. All human subject participants provided informed consent via electronic signature. As described in more detail below, participants could receive up to US $105 in gift cards for participating in the quantitative survey component of the study and US $150 in gift cards for participating in the qualitative component of the study.

### Recruitment of Study Participants

People experiencing homelessness were eligible to participate in the study if they were 18 years or older, had been approved for PSH, could be interviewed in English or Spanish, and were willing to provide informed consent. Enrollment included people experiencing homelessness who had been approved for PSH in Los Angeles County and had either been housed in the past 2 weeks or were expected to be placed in housing in 30 days. PSH placement is determined through a county-run coordinated entry system that identifies clients’ needs and matches them with available housing options. Because the system typically assigns individuals approved for PSH to specific nonprofit, community-based agencies that are ultimately charged with securing housing and supportive services, recruitment was conducted through 26 agencies. The study began recruitment in January 2021 amid a significant surge in the COVID-19 pandemic in Los Angeles County. Therefore, in-person recruitment was not a viable option and instead depended on the case managers at each program, who were already interacting with people experiencing homelessness as part of the housing placement process. The study team relied on these agencies to inform anyone approved for PSH (or rapid rehousing who would be receiving supportive services) about the study. Study staff members then coordinated a meeting with the eligible people experiencing homelessness interested in the study via phone or Zoom to complete the enrollment process. Although this process remained largely intact throughout the recruitment period, in-person recruitment occasionally occurred when COVID-19 rates were low and visitors were allowed at program sites.

Recruitment for the qualitative portion of the study involved 2 sources. The qualitative people experiencing homelessness sample was selected from those who enrolled in the quantitative portion of the study. A subsample of study participants (N=40; 20 from each PSH model type) was selected for interviews using maximum variation sampling [16] based on demographic and health characteristics (ie, Black or African American people, women, individuals with chronic disease, individuals with serious mental illness, individuals with substance use disorders or individuals aged 60 years or older), and they separately consented to participate in qualitative interviews. The provider sample (N=48; 24 from each PSH model type) was selected from housing providers who worked at agencies that were study recruitment sites. Sites were asked to identify staff members who would be willing to participate in a focus group; staff members were asked to provide verbal consent at the outset of the focus group.

### Enrollment and Quantitative Data Collection Procedures

To enroll, an agency staff member initiated the referral process for people experiencing homelessness by contacting the research recruitment team via phone, email, or text. A research recruitment team member then followed up to schedule an appointment with the potential participant to complete a screening to ensure eligibility, review the consent form, and answer any questions related to the study protocols. Case managers then distributed study phones that had been shipped to the recruitment site. Participants received a Samsung A01 Core smartphone with an unlimited data and calling plan, which allowed them to provide electronic informed consent and complete a self-administered questionnaire sent via text message. Participants were enrolled in the study once they provided informed consent by completing a web-based form that assessed study comprehension and documented their electronic signature.

Initially, participants completed a 20-minute survey upon enrollment in the study to capture basic demographic and historical information about their housing and health. Baseline outcome measures were then administered in a follow-up survey approximately 1 day later to help reduce the burden involved in completing a lengthy questionnaire at the time of enrollment. Participants received a US $15 electronic gift card incentive for completing the baseline outcome measure survey. Participants who enrolled in the study prior to moving into a housing unit were asked to complete a second baseline outcome measures survey once they moved in and received another US $15 electronic gift card incentive. After moving into PSH and completing the demographic and baseline surveys, participants received 5 monthly follow-up surveys and received a US $15 electronic gift card incentive for each completed survey. Participants could also complete surveys over the phone if they preferred to speak with a surveyor rather than self-administer the survey. Electronic gift cards were sent to an email address provided by the participant. The survey links were all sent via text message. The study team, in consultation with the LEG, opted to allow “prefer not to answer” as a response option for all historical and monthly survey questions to reduce any frustration among those who were not sure how to answer and minimize the possibility that some questions could be triggering given high rates of past trauma.

### Quantitative Measures

Patient-centered quality-of-life outcomes in this study were based on the World Health Organization’s definition of health as “a state of complete physical, mental, and social well-being and not merely the absence of disease or infirmity” [17]. Table 1 describes the study’s constructs and measures, with the exception of COVID-19–related outcomes, which are discussed in more detail subsequently.

**Table 1:** Study constructs and measures.

Construct		Variables and instruments
**Patients experiencing homelessness** **population characteristics: covariates measured at baseline only** **[18]**		
	Predisposing	Demographics (ie, age, gender, marital status, and veteran status); health beliefs (ie, knowledge of disease and health services attitudes); social structure (ie, race and ethnicity, education, employment, religion, trauma, and homelessness history); systems involvement history (eg, foster care, criminal justice, and trauma); housing preference
	Enabling	Income, insurance, benefits, case management, food insecurity, and social support
	Need	Chronic physical disease, serious mental illness, and substance use disorder; baseline measures for physical and mental health status as noted in outcomes
**Patient-centered outcomes: quality of life including physical, mental, social, and environment health, and measured monthly**		
	Life satisfaction	NIH^a^ Toolbox Item Bank v2.0 General Life Satisfaction (10 items, self-rated life satisfaction) [19]
	Physical health	PROMIS^b^ Global Health Scale Version 1.2 (PROMIS; 2 items, self-rated physical health and activities) [19]
	Mental health	PROMIS (2 items, self-rated mental health, frequency bothered by symptoms) [19]
	Social health	PROMIS (1 item, self-rated satisfaction with social activities and relationships) [19]
	Environmental health	Housing retention and Housing Environment Scale (4 items, residential satisfaction; 12 items, neighborhood quality and safety) [20]
**Health behaviors: measured monthly**		
	Substance use	Collaborating Consortium of Cohorts Producing NIDA^c^ Opportunities survey (12 items) [21]
	Health care use	COVID-19– and non-COVID-19–related past-180-day use of physical, mental, substance use, social services, and PSH^d^ support; medication adherence; ambulatory and emergency department visits and hospitalizations; barriers to care

^a^NIH: National Institutes of Health.

^b^PROMIS: Patient-Reported Outcomes Measurement Information System.

^c^NIDA: National Institute on Drug Abuse.

^d^PSH: permanent supportive housing.

### COVID-19–Related Outcomes

Given that the study project period began in October 2020, with initial recruitment starting in January 2021, our initial survey questions focused on COVID-19–related protective health behaviors that were salient at the time—namely, social distancing and handwashing. As science and the pandemic evolved, questions about COVID-19 testing and vaccines were introduced as key protective health behaviors. For COVID-19 testing on the monthly surveys, we first asked whether participants had been tested in the past 30 days and, if so, the outcome of the test. For anyone who had not been tested in the past 30 days, we asked about barriers to testing (ie, “Did you want to get tested for coronavirus [COVID-19] in the past 30 days but were unable to?”). For those who replied affirmatively, possible reasons for not getting tested included: I didn’t know how to make an appointment to be tested; I didn’t know where to go; I don’t think that I can afford the cost of the test; I didn’t have time to get tested; I am unable to travel to a testing location; I am worried about bad things happening to me or my family if I get tested (including discrimination, government policies, or social stigma).

For vaccination status, questions evolved over time. When the study began and there was only 1 approved vaccine that was not readily available, participants were asked if they “have been offered the COVID-19 vaccine?” with branch logic based on whether they said they had received the vaccine or would receive it if offered. If participants responded “no” to having received the vaccine after being offered it or to a hypothetical offer, they were prompted about the reasons for not receiving the vaccine to better understand their hesitancy. Six months into data collection, when multiple approved vaccines were more readily available, the survey was revised to first ask if a participant had received the vaccine rather than if it had been offered. Questions about the type of vaccine (brand) and the number of doses received were also added to the monthly surveys. In June 2022, the survey was revised a final time to ask new questions about receipt of COVID-19 booster vaccines using the following three uniform questions: (1) “How many doses of the COVID-19 vaccine have you received (including booster shots)? Please indicate the brand of each dose you received and the month and date when you received it”; (2) “To the best of your knowledge, have you received all COVID-19 doses or boosters that you’re eligible for?”; and (3) “In the future, how likely are you to receive another dose or booster of the COVID-19 vaccine if it’s recommended?” Participants who had already completed the study and not been asked these questions were recontacted.

### Qualitative Interview Procedures

Each member of the qualitative people experiencing homelessness sample was asked to complete 3 semistructured qualitative interviews. The first interview, conducted over the telephone and audio recorded, focused on questions concerning participants’ (1) prehousing homelessness experiences; (2) experience obtaining PSH; (3) health, social services, and social experiences prior to and after obtaining PSH; and (4) management of COVID-19–related safety and concerns. These interviews lasted approximately 30-60 minutes each. The second and third interviews were conducted 3 and 6 months after the baseline interviews and used photo elicitation interviewing (PEI), a method that uses photographs taken by participants to guide interviews [22]. This method enhances the depth of each interview and increases the capacity to identify potential topics of interest that were not part of the study’s original data collection plan. PEI is also an effective tool for building rapport, gaining access to participants’ lived experiences, and relieving the strain of direct, extended verbal questioning, which can be particularly taxing for participants with cognitive or mental disabilities [23-25]. All client PEI interviews were conducted over the phone and lasted approximately 45 minutes. Each interview was recorded and transcribed for analysis, and participants received a US $50 gift card incentive of their choice (Visa, Amazon, or Target) after each interview.

### PEI Methods

Participants were asked to use their study-issued cellphones to take photos that represent their daily lives and experiences in their housing placement for a 2-week period. There was no limit on the number of photos they could take, though they were asked to avoid taking any identifiable photos of any individuals to protect privacy. Participants were then asked to choose 5-10 of the photos that best captured their recent experiences and email or text them to the study coordinator, who then provided them to the interviewer. At the outset of each interview, interviewers asked participants to choose whichever photo they wanted to discuss first. For this photo and subsequent images, participants were asked first to describe each photo and then elaborate on how it reflects their recent experiences related to housing, health, service use, COVID-19 protection, safety, resources, relationships, personal growth, and daily functioning. Participants were also asked to come up with a title or theme that collectively described the photos they discussed, if there was anything they wanted to take a photo of but could or could not, and why, and any recommendations for improvements in services for others experiencing homelessness based on the photos. If any participant did not feel comfortable using the phone camera, they were offered the alternative of taking “mental snapshots,” in which they made a list of 5-10 things they wished to discuss and shared this list with their interviewer. For the second PEI at the 6-month follow-up, participants were asked to go through the same procedures. However, during this PEI, participants were asked to focus on how things changed for them across the different domains and compare their photos from the 2 PEIs.

### Focus Groups

Provider group interviews concentrated on learning provider perspectives on the difference between PB-PSH and SS-PSH, the strengths and weaknesses of each model in improving outcomes for people experiencing homelessness, and the impacts of the COVID-19 pandemic on PSH service delivery. Each focus group met for approximately 60 minutes and was conducted either in person or via Zoom, depending on the housing agency’s preference. As with the client interviews, staff focus groups were recorded and transcribed for analysis, and participants received a US $50 gift card incentive of their choice (Visa, Amazon, or Target).

### Quantitative Analysis

The statistical analysis will rely on bivariate and multivariate regression analysis to examine the relationship between PSH (SS-PSH vs PB-PSH) and quality of life and COVID-19–related health behaviors. For outcomes, we will use a pre-post design that treats Month 1 as the baseline and Month 6 as the endline and compares pre-post changes for SS-PSH and PB-PSH. For outcomes sensitive to monthly trajectory variation, we will use fixed-effects and random-effects models of monthly outcomes that test for interaction between the PSH model and months of exposure to each PSH type. These models will be informed by an analysis of baseline variation between SS-PSH and PB-PSH clients. We expect variations in the level of baseline morbidity. Thus, we will use both multivariate models that account for baseline differences and propensity score matching models that establish an area of common support for SS-PSH and PB-PSH comparisons. We will use calendar-month controls to account for historic changes in the ecology of health behavior, particularly in relation to rising and falling rates of COVID-19. We will also address the potential need for multilevel or mixed-effects models to account for potentially shared experiences by housing and service providers. Additional models will test for differences in housing outcomes by race, ethnicity, sex, and gender via interaction effects and stratified models. Further analysis will entail the use of a path modeling framework to understand the pathways leading from PSH types to outcomes of interest. Due to the decision to include “prefer not to answer” as an option for all questions, all analyses will account for the frequent use of this response option. In cases where “prefer not to answer” is a common or meaningful response option, such as regarding partisan political affiliation or experiences of trauma, we will include it as a response option. In cases where “prefer not to answer” appears to reflect a random nonresponse pattern, we will treat the value as missing, use multiple imputations to maintain sample size, and conduct robustness checks comparing the imputed and raw samples.

### Qualitative Data Analysis and Integration

Client semistructured interview and focus group transcripts have been imported into Dedoose [26] for analysis. Analysis was conducted using template analysis, with thematic codes defined in relation to the study questions (eg, housing process and experiences, COVID-19 experiences, and posthousing experiences), and inductively through coding the transcripts [27]. Individual team members cocoded the transcripts and discussed the codes iteratively, including collapsing, expanding, and combining codes, until consensus was reached on the codebook. Any discrepancies in codes and excerpts were discussed in team meetings and resolved in discussion with the qualitative team leaders [27]. After initial cocoding, team members coded the remainder of the transcripts using Dedoose.

PEIs will be analyzed using 2 approaches—one for the interview text and one for the photographs. Interview text will be analyzed using template analysis as previously described. Photographs are analyzed through a process of initial categorization by theme, referencing interview texts to ensure proper interpretation, and group discussion to develop an initial visual code book. Members of the team will cocode photos from 5 interviews to test the codebook’s utility and identify codes that needed further definition or clarity. Once the codebook is established, each photo will be coded independently by 2 researchers, who discussed discrepancies until they reached a consensus on all interpretations of images [22].

### Mixed Methods Analysis

As appropriate, qualitative and quantitative data will be merged to achieve several functions. First, merged data will be used to identify convergences—areas where qualitative data confirm quantitative findings, and vice versa. Second, the research team will use qualitative data complementarily to provide depth and understanding to the findings that emerge from quantitative analyses. Third, qualitative data will be used to explain and expand on findings from quantitative data. The researchers will note areas where one data source generates insights but the other does not [28].

## Results

**Figure 2:**
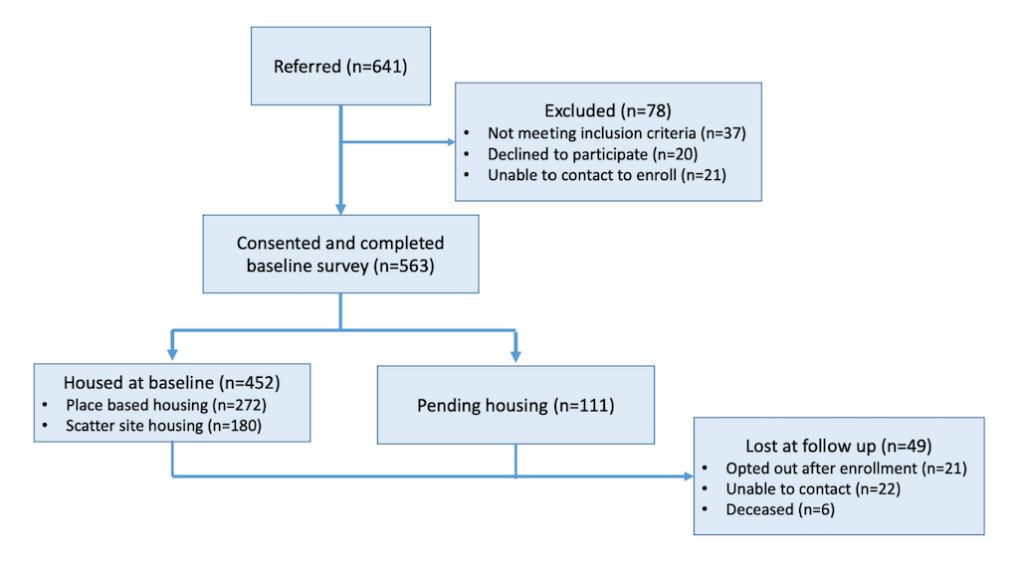
Consort enrollment diagram. PSH: permanent supportive housing.

Study recruitment was supposed to occur over 6 months starting in January 2021 but was extended due to delays in recruitment. These delays included COVID-19 delays (eg, recruitment sites shut down due to outbreaks and study team members testing positive) and delays that may have been indirectly related to the COVID-19 pandemic, including high staff turnover or recruitment sites having competing priorities. Given these delays, data collection and analysis are expected to be completed in 2023. As depicted in the consort diagram (see Figure 2), at the end of recruitment in July 2022, a total of 641 people experiencing homelessness had been referred from 26 partnering recruitment sites, and 563 people experiencing homelessness had enrolled in the study and completed a baseline demographic survey. Of the 641 referrals, 37 were not eligible to participate due to not meeting the criteria, 20 declined to participate, and our recruitment team could not reach 21 to complete enrollment. Of the 563 participants in the study, 452 had recently moved into the housing when they enrolled, with 272 placed in PB-PSH and 180 placed in SS-PSH. Another 111 participants were approved but are still waiting for housing placement. To date, 49 participants have been lost at follow-up, with 21 opting out of the study, 22 who could not be reached, and 6 participants who died.

To date, 12% of phones (70 of the initial 563 distributed) have been reported lost by participants. Of those who reported losing their phone, we lost contact with 22 participants; 28 completed the study using a replacement phone provided by the study or an alternative phone number. Redemption of electronic gift card incentives has been high. To protect respondent privacy, gift card redemption data was not linked to respondents at the personal level, but we analyzed aggregate and monthly redemption rates for all completed surveys. Among all gift cards issued, 82% were redeemed. This figure likely reflects an underestimate of overall redemption rates because the denominator double-counts individuals who were reissued a second card after failing to redeem the first. Redemption rates improved during the study, from 75% in month 1 to 86% in month 5. Because redemption data were not linked at the person level, it was impossible to discern whether redemption affected retention or response rates.

## Discussion

### Principal Findings

This paper presents the protocols from the PCHOOSE study, a mixed methods prospective longitudinal study designed to examine the comparative effectiveness of PB-PSH and SS-PSH in promoting COVID-19 protective behaviors among people experiencing homelessness. To date, the study has successfully recruited 563 participants in the study, with 452 of those participants moving into housing (272 placed in PB-PSH and 180 in SS-PSH). Another 111 participants are still waiting for housing placement. As noted, however, recruitment during the pandemic was challenging given that in-person contact was not permitted at times either by program sites or the research institutions during COVID-19 surges and high community transmission, which particularly affected homelessness programs in Los Angeles County.

To overcome recruitment challenges, flexible strategies were used that included extending the recruitment period and the distribution of cell phones with paid data plans. Although this was a viable approach to recruitment and data collection, thus far, 12% of phones have been lost. It is not clear to what extent study recruitment rates would have been different without distributing phones given the high rates of phone ownership in this population, although maintaining consistent phone service is challenging [29]. Given current recruitment numbers and retention rates that are over 90% (only 49 have been lost to follow-up), the study will be able to address a gap in the literature by considering the comparative effectiveness of PB-PSH versus SS-PSH on patient-centered quality of life, health care use, and health behaviors that reduce COVID-19 risk.

### Limitations

This study is unique in that it represents a large-scale study that started in the first year of the COVID-19 pandemic to better understand how housing programs serve as a social determinant of health for people experiencing homelessness [5]. Given this context, the study required ongoing changes to the survey instrument due to COVID-19–related outcomes, vaccine implementation, and community mitigation strategies that continued to change throughout the study period. Changing study instruments will pose challenges for analysis and also highlight the need to consider how period effects may drive changes in participant response as opposed to the time in housing that was originally planned. Other potential challenges may include discrepancies between qualitative and quantitative reporting, data richness due to qualitative phone interviews instead of in-person interviews, missing or inaccurate vaccine data, and participants failing to report on health risk behaviors such as drug use, receiving the vaccination, or other risky behaviors due to stigma. These issues will be examined and reported on after the study. We also note that this study represents a natural experiment and that people experiencing homelessness were not randomized to PSH type; further, participants were referred to the study through a case manager, which may introduce additional selection bias. Examining baseline characteristics will help determine whether there are selection differences between the 2 groups that will need to be adjusted during analyses.

### Conclusions

The PCHOOSE study protocols demonstrate that recruitment of people experiencing homelessness is possible during a global pandemic but that significant flexibility is required to conduct research with an underresourced population during major historical events, such as the COVID-19 pandemic, which reflects a period where scientific knowledge and public policies evolved rapidly. At a minimum, information gained from this project can provide insights into how people experiencing homelessness navigated the pandemic, but we may also better understand the contextual factors that contribute to COVID-19 being a housing-sensitive condition. Given that few studies have compared PB-PSH and SS-PSH, let alone amid a global health concern such as COVID-19, this study will be able to address a gap in the literature regarding how different models of PSH can affect the patient-centered quality of life, health care use, and health behaviors that reduce COVID-19 risk, which can influence future public health approaches to homelessness and infectious diseases.

## Abbreviations

LEG: lived experience group
PB-PSH: place-based permanent supportive housing
PCHOOSE: Person-Centered Housing Options, Outcomes, Services, and Environment
PEI: photo elicitation interviewing
PSH: permanent supportive housing
SAB: stakeholder advisory board
SS-PSH: scatter-site permanent supportive housing

## Data Availability

All data from this research will be made available in a public data repository (TBD), as is contractually required by the funding organization.
